# Influence of a Coaxial Electrospraying System on the n-Hexadecane/Polycaprolactone Phase Change Microcapsules Properties

**DOI:** 10.3390/ma13092205

**Published:** 2020-05-11

**Authors:** Shengchang Zhang, Yuan Chen, Christine Campagne, Fabien Salaün

**Affiliations:** 1 GEMTEX—Laboratoire de Génie et Matériaux Textiles, ENSAIT, F-59000 Lille, France; shengchang.zhang@ensait.fr (S.Z.); christine.campagne@ensait.fr (C.C.); 2 State Key Laboratory of Polymer Materials and Engineering, College of Polymer Science and Engineering, Sichuan University, Chengdu 610065, China; cyuan201431@163.com

**Keywords:** electrospraying, microencapsulation, polycaprolactone, phase change materials, n-hexadecane

## Abstract

Electrospraying is considered to be a green, high-efficiency method for synthesizing phase change microcapsules (mPCMs) for possible applications in the fields of energy storage and thermal regulation. In this study, a coaxial nozzle was used to prepare n-hexadecane/polycaprolactone (PCL) microparticles. The objectives of this study were to investigate the influence of working parameters and solutions on morphology, particle size, thermal properties and encapsulation efficiency. Thus, three theoretical loading contents in n-hexadecane (30%, 50% and 70% w/w) and two concentrations of PCL (5 and 10% w/v) were used. The structures, morphologies and thermal properties of mPCMs were characterized by optical microscopy (OM), scanning electron microscopy (SEM), differential scanning calorimeter (DSC), and thermogravimetric analysis (TGA). Spherical microcapsules with a mean diameter of 10–20 µm were prepared. The increased concentration of n-hexadecane and PCL resulted in a change in the particle size distribution from a poly-disperse to monodisperse size distribution and in a change in the surface state from porous to non-porous. In addition, higher encapsulation efficiency (96%) and loading content (67%) were achieved by the coaxial nozzle using the high core-shell ratio (70/30) and 10% w/v of PCL. The latent heat of the mPCMs reached about 134 J.g^−1^. In addition, it was also observed that the thermal stability was improved by using a coaxial system rather than a single nozzle.

## 1. Introduction

Phase change materials (PCMs) are materials that are able to store or release a large amount of latent heat with a variation of surrounding temperatures due to their phase change transition occurring in this range [1,2,3]. Among the various PCMs, n-alkanes are widely used in energy storage and thermal control because of their relatively high thermal and chemical stability, high latent heat storage capacity, appropriate phase change temperature, and low cost [4,5,6,7]. Their encapsulation in an inorganic or organic shell, or matrix, overcomes their limitations and drawbacks, such as low density, high volatilization, leaching behavior, low thermal conductivity, and flammability characteristics when used or implemented in a material structure [8,9,10]. Microencapsulation leads them to a pseudo-solid compound regardless of their physical state, which effectively protects them from the surrounding environment and prevents leakage during phase transition. This step, therefore, significantly improves the stability and durability of the PCMs. Furthermore, the increase in the surface/volume ratio of microencapsulated PCMs (mPCMs) leads to an increase in the specific contact surface area, which improves their heat transfer as well as their thermal conductivity depending on the chosen polymer shell [11,12]. Thus, mPCMs are suitable materials for delaying the increase in energy consumption and for satisfying the growing demand for thermal management in space [13], and the textile industries [14,15], industrial waste heat recovery [16,17,18], energy conservation in buildings [19,20], and solar energy use [21,22].

Recently, electrospraying as a green and high-efficiency electrohydrodynamic atomization technology, has been widely used to fabricate polymeric microcapsules [23,24,25]. Under the action of applied voltage, charged droplets will be stretched into a conical jet (or Taylor cone) at the tip of a nozzle when a balance among surface tension, electrostatic force, viscoelastic force, gravity and frictional force is obtained [26]. Coulombic repulsions in the charged jet result in the formation of droplets at the tip of the Taylor cone, which then break up into smaller droplets. Their rigidification induced by the evaporation of the solvent during the flight process allows them to be recovered in solid form on the grounded collector [27]. Electrospraying method has been recognized as offering more advantages and potential for producing microcapsules compared to other microencapsulation processes [28,29], such as in situ polymerization [30], interfacial polymerization [31], sol-gel route [32], simple or complex coacervation [33,34], phase separation and emulsification methods [35,36], or spray drying process [37]. Thus, the electrospraying method does not require the use of surfactants or toxic chemical additives, and the volume of organic solvent is limited. Furthermore, the optimization of the physico-chemical properties of the solution and the associated operating parameters make it possible to design the desired structure and/or morphologies and to obtain a narrow and monodispersed particle size distribution [38]. The desired morphology also depends on the choice of nozzle geometry. The single nozzle is mainly used to produce core-shell microcapsules [39,40] and polynuclear microcapsules [41]. The coaxial double and tri-capillary nozzle is used to manufacture yolk-like [42] and double-layered shell microcapsules [43,44].

Electrospraying is rarely used to encapsulate phase-change materials, and most researchers prefer the electrospinning method as an electro-hydrodynamic atomization technology to entrap these compounds in a core-sheath structure to form phase change nanofibres from a coaxial system [45,46,47]. Nevertheless, some attempts described by Moghaddam et al. have shown the possibility of producing n-nonadecane/sodium alginate mPCM from coaxial electrospraying [48,49,50]. The stiffening of the polymeric shell was obtained in a calcium chloride solution to produce microcapsules with an encapsulation efficiency of 59%, an enthalpy of phase change of about 88 J/g, and a mean diameter between 200 and 700 µm. Yuan et al. encapsulated n-octadecane in a polyamic acid shell using a coaxial electrospraying process [51]. For a feed ratio of 2:1 between the core and shell liquids, the encapsulation efficiency was about 85%, and the loading content of PCM in the microcapsules having a mean diameter from 1 to 2 µm was about 56%, corresponding to a phase change enthalpy about 122 J.g^−1^. Recently, Zhang et al. studied the encapsulation of n-alkane in a polycaprolactone shell using a single nozzle process [52]. Depending on the formulation of their solution, the process was carried out with an encapsulation efficiency of 70%–84%, and the resulting mPCM had an enthalpy of phase change of about 99–117 J.g^−1^. Thus, the use of the electrospraying process with a single nozzle to encapsulate a phase-change material does not achieve high encapsulation efficiency, mainly due to the loss of PCM during the flying process coupled with solvent evaporation. By using a coaxial nozzle electrospray apparatus, the solutions from the core (PCMs) and polymeric shell flow independently of the inner and outer capillaries, respectively [53]. This configuration allows a short-term limitation of the physico-chemical interactions between the core and shell compounds, which restrain the diffusion of PCMs into shell phase and induce a mono-nuclear morphology [54]. This type of morphology makes it possible to highlight some interesting functional properties of mPCM, such as their durability, shape, and thermal stabilities. Nevertheless, adjustment of the solution properties and working parameters is required for the formation of a stable Taylor cone, and thus to induce a uniform breaking process of the charged jets, which is considered to be more difficult compared to a single-nozzle electrospraying process [55,56].

Up to now, the comprehensive comparison between single and coaxial electrospraying nozzle in the encapsulation of PCM, focusing on the structures, morphologies, and thermal properties of the microcapsules was not achieved. In this study, raw n-hexadecane as the core component, and 5 or 10 w/v% of polycaprolactone (PCL) solubilized in chloroform (Chl) solution for the shell material were used in a coaxial process to investigate the effect of the selected method; the core to shell weight ratios (30/70, 50/50 and 70/30); as well as the polymer concentration on the mean diameter, particle size distribution, encapsulation efficiency, loading content, morphology, and thermal properties of the mPCMs. The structures, morphologies, and thermal properties of the obtained microcapsules were characterized by scanning electron and optical microscopies (SEM and OM), differential scanning calorimetry (DSC), and thermogravimetric analysis (TGA). The results were also compared to this obtained from a single electrospraying nozzle [52]. 

## 2. Materials and Methods

### 2.1. Materials

Polycaprolactone (PCL) having a molecular weight of about 37,000 g.mol-1 was purchased from Perstorp (Skåne County, Sweden), commercialized as CapaTM 6400, and used a shell forming material. N-hexadecane was selected as the core PCM substance and was kindly supplied by Sasol Germany Gmbh (Hamburg, Germany). Chloroform (Chl), employed as the PCL solvent, was purchased from Sigma-Aldrich Co. LLC (Saint-Quentin Fallavier, France). All the selected compounds were used without further purification.

### 2.2. Preparation of Core Liquid and Shell Liquids Used in Coaxial Nozzle Electrospraying 

Raw n-hexadecane liquid was used as the core liquid. A series of PCL electrospraying solutions at different concentrations (5 and 10 w/v %) were prepared using chloroform at 40 °C under vigorous magnetic stirring equipped with a water–jacketed reflux condenser for 1 h. Before their uses, they were cooled to room temperature.

### 2.3. Production of n-Hexadecane/PCL Microcapsules by Coaxial Nozzle Electrospraying

A CAT000002 Electrospray Starter Kit from Spraybase® AVECATS (Kildare, Ireland) was used to carry out the electrospraying process for the different working solutions. A stainless-steel coaxial nozzle with two capillaries (inner capillary: 28 gauge, inner and outer diameters of 180 and 360 µm, respectively; outer capillary: 20 gauge, inner and outer diameters of 600 and 910 µm, respectively) was used. Raw n-hexadecane liquid and PCL/chloroform solution were injected into the inner and outer capillaries, respectively. The flow rates of the core or shell solutions were controlled by the syringe pump connected to a computer, and different theoretical core to PCL ratios (00/100, 30/70, 50/50, and 70/30 by weight) were achieved by changing the feed ratio between the core and shell liquids (Table 1). The applied voltage between the nozzle and collector was set at 8.25 kV. Samples were collected on a grounded copper plate covered with glass slides and an aluminum foil for OM and SEM analyses, respectively, and with a metal dish for the other characterization. The collection times were adjusted to 3, 10, and 300 min, respectively. All the containers were placed at 17 cm from the tip of the nozzle, and during the various experiments, temperature and relative humidity were kept constant at 25 °C and 45%, respectively.

### 2.4. Morphological Characterization of Electrosprayed mPCM

The surface morphology of the microparticles was observed with the help of a scanning electron microscopy (SEM), an Inspect F50 from FEI Company (USA)) at an accelerated voltage of 20 kV. Prior to analyses, the samples were coated in gold. Particle size determination was carried out on at least 200 microparticles taken from optical microscopy (OM) images on a Motic BA410 (Narcelona, Spain) equipped with a Moticam 5 digital camera (Barcelona, Spain). The images were captured using Motic image plus 3.0 software. Image J software was also used to analyze all the microscopic images; the data was compiled in a spreadsheet (Microsoft Excel 2011, Microsoft Company, Redmond, WA, USA) for further processing.

### 2.5. Thermal Analyses of Electrosprayed mPCM

The differential scanning calorimetry (DSC) was used to point out the thermal transitions and the associated latent heat values (melting (ΔHm) and crystallization (ΔHC) enthalpies); and the phase change temperatures such as the onset temperatures of solid-liquid and liquid-solid transitions (Tonset), end temperatures of phase transitions (Tend), and maximum temperatures of melting (Tm) and crystallization (Tc). The experiments were performed with 3–8 mg of the sample (n-hexadecane, PCL microparticles, and microcapsules) on a DSC 3+ from Mettler Toledo (Columbus, OH, USA) piloted on PC with STARe software, under a nitrogen atmosphere, at a heating and cooling rate of about 5 °K.min^−1^. The temperature range considered in this study was from −20 °C and 80 °C, and two thermal cycles were done. The results presented were obtained by averaging the data collected from a series of three independent experiments, during the second cycle.

The loading content (LC, Equation (1)), and the encapsulation efficiency (EE, Equation (2)) were calculated according to the measured enthalpy [34].

(1) $$LC=\frac{\Delta H_{mPCM}}{\Delta H_{PCM}}$$

(2) $$EE=\frac{LC}{LC_{th}}$$

where, Δ*H_mPCM_* and Δ*H_PCM_* are the melting enthalpies of microparticles and *n*-hexadecane (199 J·g^−1^), respectively. *LC_th_* is the theoretical loading content.

The crystallinity index (*Χc*) of the PCL in the particles (measured and theoretical) was obtained from Equations (3) and (4).

(3) $${Χ}_{c\left(m\right)}=\frac{\Delta H_{m\;\left(PCL\;phase\;in\;mPCM\right)}}{\Delta H_{m\left(PCL\right)}^{0}\left(1-w_{m\;\left(m\right)}\right)}\times 100$$

(4) $${Χ}_{c\left(th\right)}=\frac{\Delta H_{m\;\left(PCL\;phase\;in\;mPCM\right)}}{\Delta H_{m\left(PCL\right)}^{0}\left(1-w_{m\;\left(th\right)}\right)}\times 100$$

where, the indices m and th represent the measured and theoretical weight; Δ*H_m_* is the specific melting heat; Δ*H^0^_m_* is the theoretical specific heat of 100% crystalline PCL, which was taken as 139.5 J·g^−1^; and *w_m_* was the weight fraction of PCM in the samples.

The thermogravimetric analysis (TGA) was used to determine the thermal stability of all the samples. It was carried out on a TGA 3+ (Mettler Toledo, Columbus, OH, USA) at a flow rate of 50 mL·min^−1^. For each experiment, a sample of 3 to 8 mg was used, and a heating rate of 10 °K. min^−1^ was applied from 20 to 700 °C. The variation in percentage weight (TG) and the calculated derivative percentage weight (DTG) curves were recorded and calculated for each test.

## 3. Results and Discussion

### 3.1. Size and Morphology of Electro-Sprayed mPCM

**Figure 1:**
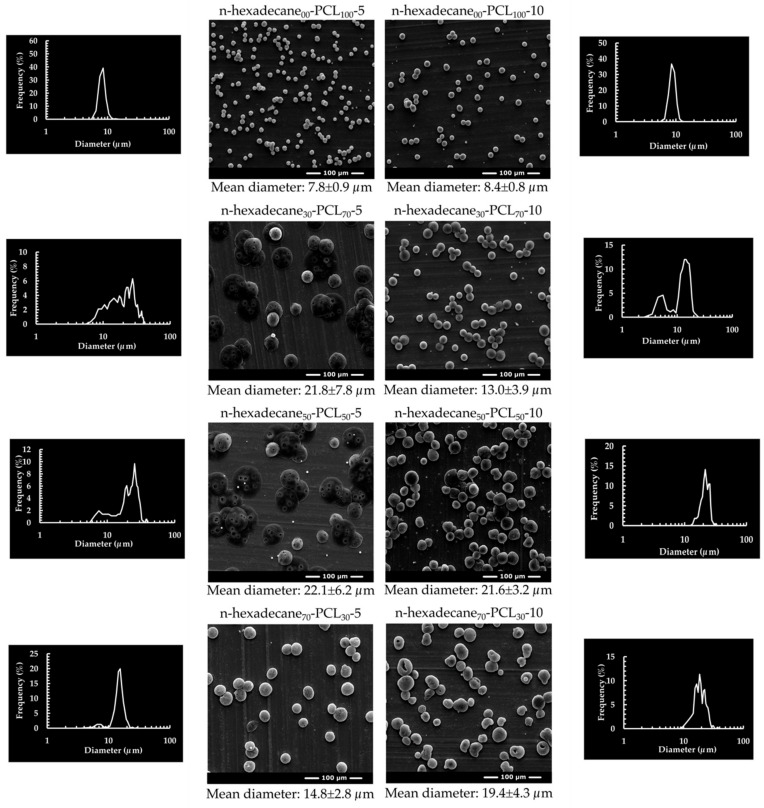
Scanning electron microscopy (SEM) images of the various microparticles and their particle size distribution.

**Figure 2:**
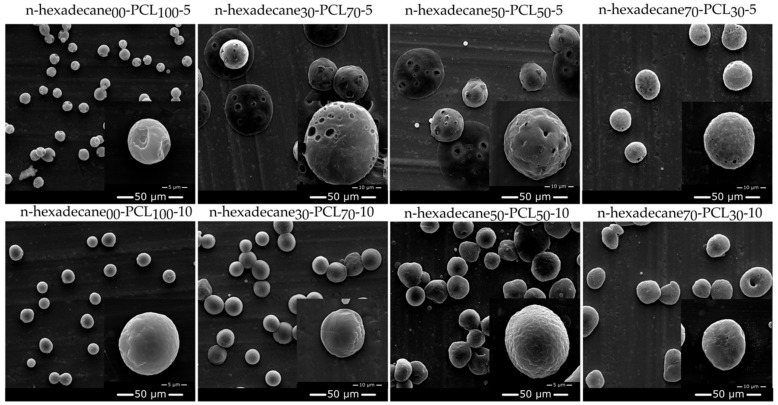
Surface state and morphologies of PCL, and PCM-PCL microparticles obtained from SEM analyses.

The mean diameter, size distribution, and morphology of electrosprayed mPCMs from the different formulations were characterized by OM and SEM observations (Figure 1 and Figure 2, Table 2). The polymer concentration does not influence the particle size properties of n-hexadecane-free PCL microparticles. They have a mean diameter between 7 and 9 µm and narrow size distribution. The addition of the n-hexadecane solution results in an increase in the mean diameter, which varies according to the amount of PCM and the concentration of the PCL solution. Thus, at 5 w/v % for levels of 30 and 50% n-hexadecane, the mean diameter is 22 µm, with a wide size distribution, whereas for 70%, the diameter decreases and the size distribution is relatively narrow. 

The phenomenon is different for 10 w/v % in PCL since an increase of 30%–50% leads to an increase in mean diameter, which is approximately the same for 70%. These different variations are related to the low electrical conductivity of the n-hexadecane solution, which limits its polarization under the action of electrostatic forces. The formation of the Taylor cone is mainly related to the physico-chemical properties of the PCL solution. It allows the realization of the cone-jet mode at the tip of the coaxial nozzle, due to the stabilization of the n-hexadecane solution by the viscoelastic forces and the interracial interactions of this solution [36]. Nevertheless, obtaining a broader size distribution is one of the consequences of the instability of the breaking process of the charged droplets, due to the unstable coulombic repulsions. Indeed, they lead to different encapsulated quantities of n-hexadecane within the particles. 

Some PCL droplets entrapped more n-hexadecane to form bigger microparticles, while others trapped less n-hexadecane to form smaller ones. A higher amount of n-hexadecane leads to a progressive narrowing of the size distribution of the microcapsules, which is linked to a decrease in the quantity of the small ones and an increase of the large ones. Besides, the increase in the PCL solutions (or PCL concentration) allows stabilizing the n-hexadecane core solution during the Taylor cone formation and the breakup process. Therefore, it leads to the obtention of a mono-dispersed distribution, especially when the core to shell proportion is higher than 30:70.

A higher amount of n-hexadecane leads to a progressive narrowing of the size distribution of the microcapsules, which is linked to a decrease in the quantity of the small particles and an increase of the large particles. Besides, the increase in the PCL solutions (or PCL concentration) allows stabilizing the n-hexadecane core solution during the Taylor cone formation and the breakup process. Therefore, it leads to the obtention of a mono-dispersed distribution, especially when the core to shell proportion is about 30:70. For single nozzle electrospraying, microcapsules with a mono-disperse distribution are obtained regardless of the amount of n-hexadecane used [52]. Indeed, the presence of the solvent increases the electrical conductivity, which leads to better stabilization of the Taylor cone and facilitates the breaking process. This phenomenon is only achieved for the higher core flow rates or higher PCL concentrations when the coaxial system is used.

### 3.2. Surface State of the Electrosprayed mPCM

The PCL concentration has a direct influence on the surface state of the electrosprayed microparticles (Figure 2). Thus, non-porous microparticles having wrinkled and smooth surface states are obtained with 5 and 10 w/v % of PCL solution (samples n-hexadecane_00_-PCL_100_-5 and n-hexadecane_00_-PCL_100_-10), respectively. The deformation of the electrosprayed droplets is mainly related to the physico-chemical properties of the solvent used. In the case of chloroform, its high vapor pressure induces quick evaporation during the flying process of the electrosprayed droplets from the nozzle to the collector. A higher PCL concentration leads to a higher solution viscosity, and the macromolecular chain entanglements act as a barrier to the solvent evaporation process. Thus, 10 w/v % of the PCL solution allows for obtaining spherical microparticles with a smooth surface state [50].

The presence of n-hexadecane in the internal capillary of the coaxial nozzle induces some changes in the formation of microparticles at low concentrations of PCL (samples labelled n-hexadecane_x_-PCLy-5). They appear to be porous with a smooth outer polymer film. In the first step of the process, the solvent diffuses into the core substance, resulting in the rapid solidification of the outer PCL matrix. During the flight process, the chloroform trapped in the inner core of the microparticles diffuses through the macromolecular chain network of the PCLs and causes tiny holes on the surface of the particles. This phenomenon is less pronounced when the amount of core substance increases or the content of the outer phase decreases. Thus, the core substance acts as a barrier to solvent evaporation in the coaxial configuration. The use of a higher concentration of PCL leads to the formation of smooth and spherical microparticles for a small amount of n-hexadecane (n-hexadecane_30_-PCL_70_-10 and n-hexadecane_50_-PCL_50_-10). On the other hand, when the amount of n-hexadecane increases or the PCL decreases, more specially for the sample n-hexadecane_70_-PCL_30_-10, the formation of a porous structure is observed, due to the competition between the formation of the solid polymer shell, the evaporation of the solvent, and the break-up process. In addition, the spherical form takes on an ovoid shape.

### 3.3. Phase Change Properties of Electrosprayed mPCM

**Figure 3:**
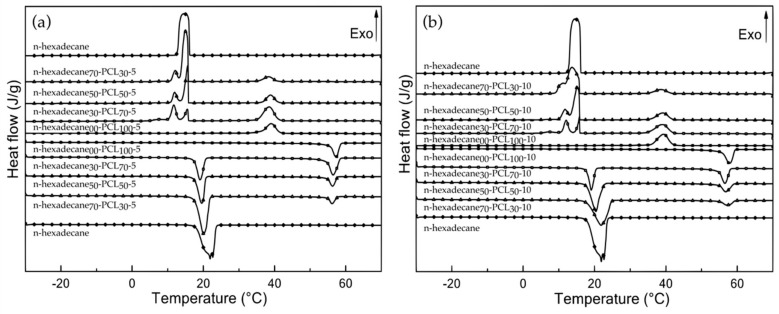
DSC thermograms of raw n-hexadecane, neat PCL microparticles and a series of n-hexadecane/PCL microcapsules obtained from PCL concentrations at 5 (**a**) and 10 (**b**) w/v % with different loading ratios between n-hexadecane and PCL.

Phase change properties of the electrosprayed neat PCL microparticles, raw n-hexadecane, and n-hexadecane/PCL particles, such as phase change temperatures and their related latent heats, have been determined by DSC measurements and are summarized in Table 3. The latent heat of melting and crystallization, and their related temperatures for the raw n-hexadecane were found at about 199 J.g^−1^, 17.9 °C, and 16.2 °C respectively. Those of PCL microparticles, namely PCL_100_-5 and PCL_100_-10, have been measured at 61.6 J.g^−1^ (58.0 J.g^−1^), 55.6 °C, and 41.4 °C (41.8 °C) respectively. Thus, the concentration of PCL in the shell solution used changes the crystallinity index to some extent, since it is about 44.2% and 41.6% for the samples prepared from 5% and 10% by weight, respectively. Furthermore, the onset melting and crystallization temperatures change little (0.5 °C) or not at all depending on the concentration used. DSC thermograms of microencapsulated n-hexadecane show the thermal transitions of the PCM and PCL (Figure 3).

During the microencapsulation process, due to the variation in viscous forces and surface tension between the two phases, n-hexadecane molecules were dispersed in the pores of the PCL matrix and also located in the inner core of the particles. Therefore, the PCM co-exist in two “states”, the bulk n-hexadecane state and the confined n-hexadecane state. The difference in the free energy of these two phases leads to the presence of an intermediate RI phase, which is not present in the raw state of this molecular solid [57]. Thus, the crystallization of the confined and bulk n-hexadecane in the microparticles occurs in two independent transformation steps, which is observed by the presence of two differential scanning calorimetry transformation peaks [58].

Increasing n-hexadecane to PCL by 30 to 70 wt. % in the working solution results in an increase in the enthalpy of fusion (and crystallization) of 52 to 115 J.g^−1^, and 46 to 134 J.g^−1^, for 5 and 10 wt. % PCL, respectively. These values also correspond to an increase in the loading content of n-hexadecane in mPCMs from 26.2% to 58.0%, and from 23.4% to 67.4%, which is lower than the theoretical content. The variations are relatively lower for the 10% PCL solution by weight, suggesting that the viscosity of the solution acts as a key parameter in the encapsulation process, in relation to the encapsulation efficiency values (EE %). The lower encapsulation efficiency, or "phase change efficiency", determined for the samples n-hexadecane_30_-PCL_70_-5 and n-hexadecane_30_-PCL_70_-10, can be related either to the unstable breakup process of the mixed droplets or to the trapping of n-hexadecane molecule chains in the PCL macromolecular, such as a plasticizing agent. In this configuration, the PCM chains are not able to organize themselves and they cannot have a phase change transition. The increase of n-hexadecane in the system allows preventing the diffusion of the molecular chains in the PCL matrix, which allows the n-hexadecane molecular chains to organize themselves and therefore induces phase change transitions. The loading content in n-hexadecane in the electrosprayed microparticles when the 10 w/v % was used in the coaxial nozzle system is close to the theoretical ones. Furthermore, the latent heat of phase change reached 134 J.g^−1^, for the sample labeled n-hexadecane_70_-PCL_30_-10, which shows that more than 96% of n-hexadecane was entrapped in the PCL matrix and may undergo a phase change transition with the temperature changes. Thus, apart from the value for 30% n-hexadecane, the encapsulation efficiency values are always higher for processes prepared from a 10% PCL solution rather than a 5% PCL solution. Also, for all systems, the encapsulation efficiency reaches a maximum value for an n-hexadecane charge amount of 50% by weight at 92.5% and 98.7% for 5% and 10% by weight PCL solutions, respectively. Besides, the loading content has no significant effect on the onset phase change transition temperatures, which are slightly lower (0.2 or 0.3 °C) than the raw n-hexadecane ones.

**Figure 4:**
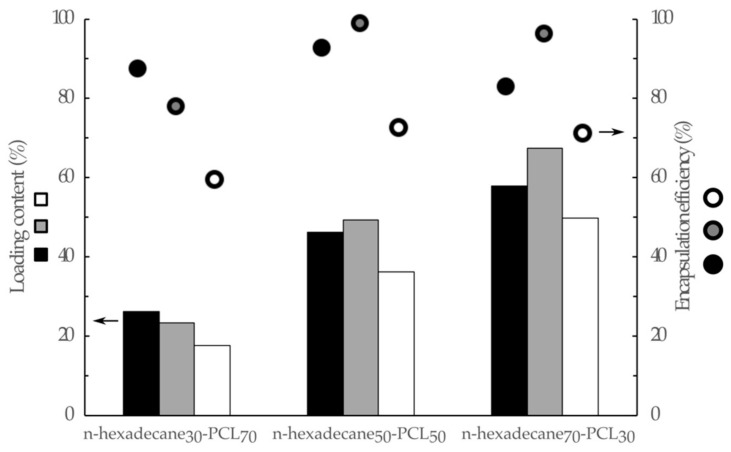
Influence of the nozzle system and the concentration of the PCL solution on the loading content and encapsulation efficiency (black—coaxial nozzle—5 w/v % PCL; grey—coaxial nozzle—10 w/v % PCL; white—single nozzle—10 w/v % PCL).

One of the main interests in the use of a coaxial nozzle system to encapsulate phase change material is related to the increase in the efficiency of the system. Thus, with regard to our previous study [52], it is noticed that the encapsulation efficiency as well as the loading content of n-hexadecane in mPCMs depend on the type of nozzle and the solutions used. The encapsulation efficiency and loading content of n-hexadecane in mPCM obtained from a coaxial nozzle electrospraying system were found to be higher than with a single nozzle one at 10 w/v % PCL solution. Furthermore, the data gathered in Figure 4 illustrated also that the values obtained with a single nozzle at 10 w/v % are lower than at 5 w/v % with a coaxial system. The use of the coaxial system allows reaching a loading content of about 58.0% or 67.4% for 5% and 10 w/v % of PCL compared to 49.9% for the single nozzle. The encapsulation efficiency corresponding to these values increased to 10 or 25 points compared to that obtained for the single system.

The loss of n-hexadecane is related to the evaporation of the solvent during the flying process in the electrospray system. In the single nozzle configuration, n-hexadecane and PCL are mixed in the same solvent solution before being used in the experiment, resulting in chemical interactions between the three compounds, depending on the solubility parameters. On the one hand, during the flight process from the nozzle to the collector, n-hexadecane molecules present in the solvent phase can diffuse directly into the air if the concentration of PCL in the charged droplets is too low. On the other hand, in the coaxial configuration, the n-hexadecane-solvent is the inner solution and the PCL/solvent is the outer one. Thus, during the process, the solvent of the inner solution can diffuse at the droplet/air interface, and the presence of PCL macromolecular chains prevents diffusion of n-hexadecane due to molecular interactions. As a result, the n-hexadecane molecules are entirely entrapped by the PCL chains, leading to the formation of a core/shell structure due to PCL stiffening, and if the concentration of the PCL solution is sufficient, smooth, non-porous particles are obtained. The morphology of the microparticles also depends on the type of nozzle used.

The electrospraying process also affects the crystallinity of the PCL matrix. The calculated crystallinity index of PCL in the mPCM is influenced by the PCL concentration and n-hexadecane content used, which is correlated with the crystallization rate during the electrospraying process. For each sample, the measured crystallinity index values (*Χ_c(_*_m)_) are lower than the calculated (theoretical) values (*Χ_c(_*_th)_), which is similar to the use of a single nozzle [52]. The variation in the crystallinity index is related to the physico-chemical phenomena occurring in the earlier stage of the electrospraying process. Furthermore, an increase of the PCL concentration leads to a decrease of the crystallinity index from 44.2% to 41.6% due to quick stiffening during the flying process, which limits the organization of the macromolecular chains (Table 3). In a coaxial system, the n-hexadecane and PCL molecular chains are not mixed; therefore, the PCM chains are less entangled in the PCL ones during the solvent evaporation. Nevertheless, the presence of PCM molecules reduces the mobility of PCL macromolecular chains and thus limits their availability for crystallization. It results in a more amorphous matrix [59,60,61,62]. The presence of n-hexadecane in the particles leads to a decrease in the onset melting temperature of about 1 °C, whereas this influence is less marked for the crystallization phenomena. On the one hand, it is noticed that the phase change enthalpy of the PCL is correlated to the amount or loading content of n-hexadecane, and therefore, decreases with the increase of PCM in the solution. On the other hand, at low PCL concentration when the n-hexadecane content increases, the amorphous phase of the obtained particles also increases, whereas at 10 w/v % no changes are observed. Furthermore, the crystallinity index of the mPCM is higher than the blank PCL particles one.

### 3.4. Thermal Stability of Electrosprayed mPCM

**Figure 5:**
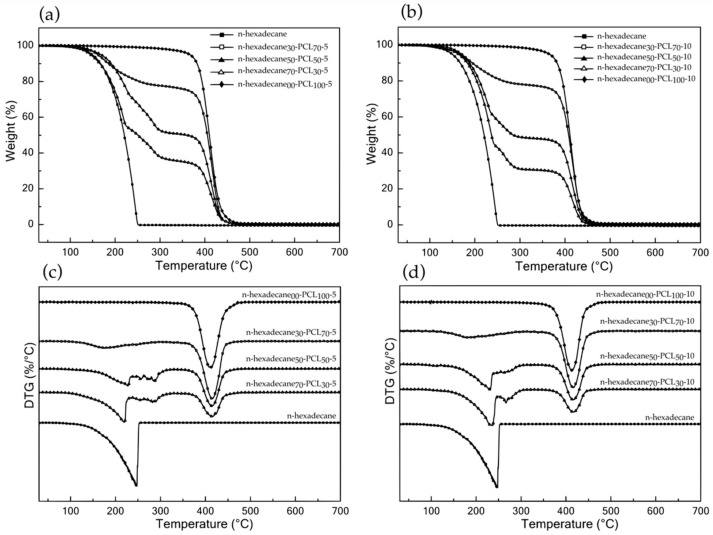
TG and DTG curves of raw n-hexadecane and n-hexadecaneX-PCLY-5 w/v % (**a**,**c**) and n-hexadecaneX-PCLY-10 w/v % (**b**,**d**), respectively.

The thermal stability of raw n-hexadecane, neat PCL microparticles, and mPCM was analyzed by TGA. The various thermograms, as well as the calculated DTG curves, are shown in Figure 5, and most of the primary data are summarized in Table 4. The thermal behavior of n-hexadecane displays single step degradation under N_2_ atmosphere and is characterized by an onset temperature at 5% weight loss (T_5%_), which starts at 149.3 °C; the maximum decomposition step of the n-alkane backbone takes place at 247.3 °C; and the maximum rate of decomposition is about 2.1 % °C^−1^. At over 280 °C, no residue was observed. The degradation of blank PCL microparticles, regardless of the concentration of the initial solution, occurs in a single step degradation between 360 and 460 °C, with a maximum degradation rate of about 2.1% or 2.2% °C^−1^ at 413 °C. The concentration in PCL in the initial solution has little influence on these thermal properties.

N-hexadecane-based microparticles exhibit a two-stage degradation, i.e. (i) from 150 to 300 °C, attributed to the degradation of n-hexadecane, and (ii) from 350 to 500 °C corresponding to that of the PCL matrix. The first stage can be divided into two main steps, i.e. (i) evaporation of the leakage core materials from 150 to 200–250 °C depending on the n-hexadecane / PCL weight ratio, and (ii) the last step has been attributed to n-hexadecane from within the microparticles or trapped in the macromolecular chains of the PCL, which evaporate completely at about 280–300 °C. The increase in the onset temperature of the first stage indicated the protective effect of the shells, which enhanced the thermal stability of the core materials. The shape of the TGA (and DTG) curve is related to the different phenomena that occur during the test due to the change in the physical state of the materials. The increase in temperature up to 62 °C causes the melting of the PCL; therefore, from this point on, all compounds are in a liquid state. The lower density of n-hexadecane allows it to diffuse to the surface of the crucible and thus initiate its degradation or evaporation from 150 °C onwards. However, as the loading content of n-hexadecane increases, these molecules become trapped in the liquid matrix of the PCL, and most of the chains become entangled. Thus, the diffusion of molecular chains has been delayed or shifted to higher temperatures since the presence of PCL acts as a physical barrier to the thermal degradation of n-hexadecane. Besides, the decrease in the PCL content leads to an increase and a decrease in the maximum rate of degradation in the first step and in the second step, respectively.

**Figure 6:**
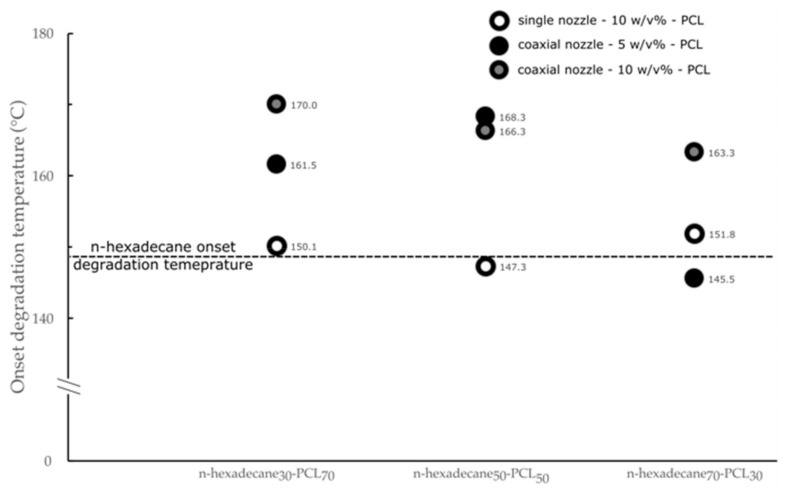
Influence of the electrospraying process on the thermal degradation of the mPCMs.

The variations of the onset degradation temperature are another example of the effect of the electrospraying system on the properties of the microparticles (Figure 6). On the one hand, mPCMs obtained from a single nozzle have an onset degradation temperature close to the raw-hexadecane ones, whatever the n-hexadecane to PCL ratio used. On the other hand, the measured T_5%_ is higher for microparticles obtained from a coaxial nozzle, except for the sample labeled n-hexadecane_70_-PCL_30_-5. This difference is related to the change in the particle morphology. It was postulated that the use of a single nozzle leads to the formation of a material structure, in which tiny droplets of n-hexadecane are dispersed in the PCL matrix [50]. The use of the 10 w/v % of PCL solution from coaxial nozzle electrospraying allows for obtaining higher onset degradation temperatures, which tends to decrease with the increase of the n-hexadecane to PCL weight ratio. For the sample n-hexadecane_70_-PCL_30_-10, T_5%_ was found 14 °C higher than this of raw n-hexadecane, underlining the protective barrier of the PCL during the degradation process, which prevents the diffusion of the PCM molecule. Thus, based on the TGA and DSC results, the use of the 10 w/v% PCL solution leads to the formation of a core-shell structure, such as a microcapsule. The decrease of the T_5%_ value with the increase of n-hexadecane content is related to the decrease of the thickness of the PCL shell. The use of the 5 w/v% PCL solution in a coaxial system induces the formation of a morphology intermediate of the two other cases, such as a salami-like structure. At higher PCL content, the n-hexadecane core materials are entirely confined in the polymeric matrix. The increase of core content leads to the formation of a porous structure due to the mixing of both solutions and the solvent evaporation during the flying process. When the ratio n-hexadecane/PCL reaches 70/30, the thermal stability of the blend PCM and PCL macromolecular chains constituting a part of the matrix structure is too weak to protect the dispersed n-hexadecane from its thermal degradation. 

## 4. Conclusions 

n-hexadecane was successfully encapsulated in a PCL matrix with a coaxial nozzle electrospraying system. The use of a 10 w/v % PCL solution allows forming a Taylor cone to stabilize the breakup process of double-component liquid, and therefore, monodispersed microcapsules with a mean diameter of about 20 µm are obtained for an initial loading content of n-hexadecane of about 50% or 70%. The concentration of the PCL solution and/or the initial content of n-hexadecane also affect the surface morphology of the obtained microparticles. Thus, the increase from 5 to 10 w/v % of the PCL solution, as well as the n-hexadecane when a 5 wt % of PCL was used, changed a porous surface state to a non-porous one. The use of a coaxial system also allows increasing a higher encapsulation efficiency compared to a single nozzle process. Furthermore, higher loading content of n-hexadecane was also achieved. Thus, when 70% n-hexadecane was introduced in 10 w/v % PCL solution, the encapsulation efficiency of n-hexadecane reached 96.3%, and correspondingly, the active loading content of n-hexadecane reached 67.4%. This content allows storing more than 130 J.g^−1^ of latent heat via its liquid-solid phase transition. The presence of PCL surrounding the n-hexadecane phase enhances its thermal stability. Furthermore, the morphology of the microparticles can be adjusted depending on the initial PCL and n-hexadecane contents in the formulation, as expected from the TGA and DSC tests. Thus, a lower n-hexadecane to PCL ratio leads to a matrix morphology, whereas the higher amount in n-hexadecane allows obtaining either a salami-like or a core/shell structure. The use of a coaxial system allows for improving the encapsulation efficiency and loading content of n-hexadecane compared to a single nozzle electrospraying. Stable cone-jet mode and stable breakup process of charged droplets are more comfortable to control for a single nozzle than for a coaxial electrospraying, where the outer physico-chemical properties play a crucial role. The formation of microparticles from an electrospraying process is an excellent alternative to other physical or chemical microencapsulation methods and is a high-efficiency choice for energy storage and thermal regulation system.

## Figures and Tables

**Table 1:** Solutions and process flow rates used for the mPCM synthesis.

Sample Label ^a^	PCL Concentration (w/v %)	Shell Flow Rate (mL/h)	Core Flow Rate (mL/h)
n-hexadecane_00_-PCL_100_-5	5	1.00	0.00
n-hexadecane_30_-PCL_70_-5	5	1.00	0.03
n-hexadecane_50_-PCL_50_-5	5	1.00	0.07
n-hexadecane_70_-PCL_30_-5	5	1.00	0.17
n-hexadecane_00_-PCL_100_-10	10	1.00	0.00
n-hexadecane_30_-PCL_70_-10	10	1.00	0.06
n-hexadecane_50_-PCL_50_-10	10	1.00	0.14
n-hexadecane_70_-PCL_30_-10	10	1.00	0.33

^a^ Samples are labelled as n-hexadecane_X_-PCL_Y_-Z, with X the PCM content (%), Y the PCL content (%), and Z the PCL concentration in chloroform solution (w/v%).

**Table 2:** The mean diameter and size distribution of obtained microparticles.

Shell Liquid Concentration	n-Hexadecane_00_-PCL_100_	n-Hexadecane_30_-PCL_70_	n-Hexadecane_50_-PCL_50_	n-Hexadecane_70_-PCL_30_
5 wt %	7.8±0.9 µm	21.8±7.8 µm	22.1±6.2 µm	14.8±2.8 µm
10 wt %	8.4±0.8 µm	13.0±3.9 µm	21.6±3.2 µm	19.4±4.3 µm

**Table 3:** Thermal properties of n-hexadecane, neat PCL microparticles and a series of n-hexadecane/PCL microcapsules obtained from different PCL concentrations at three loading ratios between n-hexadecane and PCL.

Sample Label		Latent Heat (J/g)		T_onset_ (°C)		LC (%)	EE (%)	X_c(th)_ (%)	X_c(m)_ (%)
n-hexadecane	heating	199.4	-	17.9	-	-	-	-	-
	cooling	195.9	-	16.2	-	-	-	-	-
n-hexadecane_00_-PCL_100_-5	heating	-	61.6	-	55.6	-	-	44.2	-
	cooling	-	59.1	-	41.4	-	-	-	-
n-hexadecane_30_-PCL_70_-5	heating	52.2	56.9	17.5	54.7	26.2	87.3	58.5	55.5
	cooling	51.1	56.7	15.9	41.0	-	-	-	-
n-hexadecane_50_-PCL_50_-5	heating	92.0	35.0	17.6	54.8	46.2	92.5	50.4	46.8
	cooling	90.4	35.7	15.9	41.0	-	-	-	-
n-hexadecane_70_-PCL_30_-5	heating	115.5	15.8	17.6	54.9	58.0	82.9	37.9	27.1
	cooling	112.9	18.2	15.9	40.6	-	-	-	-
n-hexadecane_00_-PCL_100_-10	heating	-	58.0	-	55.6	-	-	41.6	-
	cooling	-	56.0	-	41.8	-	-	-	-
n-hexadecane_30_-PCL_70_-10	heating	46.5	49.7	17.8	54.7	23.4	77.9	50.9	46.5
	cooling	45.5	50.0	16.0	41.9	-	-	-	-
n-hexadecane_50_-PCL_50_-10	heating	98.3	32.7	17.7	54.8	49.4	98.7	46.9	46.1
	cooling	97.5	33.2	16.0	41.8	-	-	-	-
n-hexadecane_70_-PCL_30_-10	heating	134.1	20.5	17.7	54.9	67.4	96.3	49.0	45.1
	cooling	130.3	21.1	16.0	42.0	-	-	-	-

**Table 4:** Thermogravimetric data of raw n-hexadecane, neat PCL microparticles and a series of n-hexadecane/PCL microcapsules obtained from two PCL concentrations with different loading ratios between n-hexadecane and PCL.

Sample	Initial Degradation Temperature – T_5%_ (°C)	First Step			Second Step		
		Weight Loss (100–350 °C ) (%)	Maximum Degradation Temperature (°C)	Maximum Degradation Rate (%/°C)	Weight Loss (350–500°C) (%)	Maximum Degradation Temperature (°C)	Maximum Degradation Rate (%/°C)
n-hexadecane_00_-PCL_100_-5	366.8	3.4	-	-	96.1	412.7	2.1
n-hexadecane_00_-PCL_100_-10	364.7	3.6	-	-	95.8	413.8	2.2
n-hexadecane	149.3	99.3	247.3	2.1	-	-	-
n-hexadecane_30_-PCL_70_-5	161.5	24.0	177.7	0.2	75.5	416.3	1.9
n-hexadecane_50_-PCL_50_-5	168.5	49.3	227.0	0.6	50.0	414.7	1.2
n-hexadecane_70_-PCL_30_-5	145.5	64.4	218.3	1.0	34.8	415.0	0.8
n-hexadecane_30_-PCL_70_-10	170.0	23.1	180.5	0.2	76.4	415.7	1.8
n-hexadecane_50_-PCL_50_-10	166.3	51.8	231.1	0.8	47.6	416.5	1.2
n-hexadecane_70_-PCL_30_-10	163.3	69.3	236.8	1.2	30.1	416.5	0.7

## References

[1] Nazir H., Batool M., Osorio F.J.B., Isaza-Ruiz M., Xu X., Vignarooban K., Phelan P., Inamuddin, Kannan A.M. (2019). Recent developments in phase change materials for energy storage applications: A review. Int. J. Heat Mass Transf..

[2] Safari A., Saidur R., Sulaiman F.A., Xu Y., Dong J. (2017). A review on supercooling of Phase Change Materials in thermal energy storage systems. Renew. Sustain. Energy Rev..

[3] Souayfane F., Fardoun F., Biwole P.-H. (2016). Phase change materials (PCM) for cooling applications in buildings: A review. Energy Build..

[4] Qiu X., Song G., Chu X., Li X., Tang G. (2013). Microencapsulated n-alkane with p(n-butyl methacrylate-co-methacrylic acid) shell as phase change materials for thermal energy storage. SoEn.

[5] Qiu X., Lu L., Wang J., Tang G., Song G. (2015). Fabrication, thermal properties and thermal stabilities of microencapsulated n-alkane with poly(lauryl methacrylate) as shell. Thermochim. Acta.

[6] Döğüşcü D.K., Kızıl Ç., Biçer A., Sarı A., Alkan C. (2018). Microencapsulated n -alkane eutectics in polystyrene for solar thermal applications. SoEn.

[7] Salaün F., Devaux E., Bourbigot S., Rumeau P. (2009). Development of Phase Change Materials in Clothing Part I: Formulation of Microencapsulated Phase Change. Text. Res. J..

[8] Cao F., Yang B. (2014). Supercooling suppression of microencapsulated phase change materials by optimizing shell composition and structure. ApEn.

[9] Qiu X., Li W., Song G., Chu X., Tang G. (2012). Microencapsulated n-octadecane with different methylmethacrylate-based copolymer shells as phase change materials for thermal energy storage. Energy.

[10] Alva G., Lin Y., Liu L., Fang G. (2017). Synthesis, characterization and applications of microencapsulated phase change materials in thermal energy storage: A review. Energy Build..

[11] Wang T., Wang S., Geng L., Fang Y. (2016). Enhancement on thermal properties of paraffin/calcium carbonate phase change microcapsules with carbon network. ApEn.

[12] Salaün F., Devaux E., Bourbigot S., Rumeau P. (2008). Preparation of multinuclear microparticles using a polymerization in emulsion process. J. Appl. Polym. Sci..

[13] Mondal S. (2008). Phase change materials for smart textiles—An overview. Appl. Therm. Eng..

[14] Onder E., Sarier N., Cimen E. (2008). Encapsulation of phase change materials by complex coacervation to improve thermal performances of woven fabrics. Thermochim. Acta.

[15] Shin Y., Yoo D.-I., Son K. (2005). Development of thermoregulating textile materials with microencapsulated phase change materials (PCM). II. Preparation and application of PCM microcapsules. J. Appl. Polym. Sci..

[16] Lin Y., Jia Y., Alva G., Fang G. (2018). Review on thermal conductivity enhancement, thermal properties and applications of phase change materials in thermal energy storage. Renew. Sustain. Energy Rev..

[17] Xia M., Yuan Y., Zhao X., Cao X., Tang Z. (2016). Cold storage condensation heat recovery system with a novel composite phase change material. ApEn.

[18] Jia J., Lee W.L. (2015). Experimental investigations on using phase change material for performance improvement of storage-enhanced heat recovery room air-conditioner. Energy.

[19] Xia Y., Zhang X.-S. (2016). Experimental research on a double-layer radiant floor system with phase change material under heating mode. Appl. Therm. Eng..

[20] Johra H., Heiselberg P. (2017). Influence of internal thermal mass on the indoor thermal dynamics and integration of phase change materials in furniture for building energy storage: A review. Renew. Sustain. Energy Rev..

[21] Sharma R.K., Ganesan P., Tyagi V.V., Metselaar H.S.C., Sandaran S.C. (2015). Developments in organic solid–liquid phase change materials and their applications in thermal energy storage. Energy Convers. Manag..

[22] Wang Z., Qiu F., Yang W., Zhao X. (2015). Applications of solar water heating system with phase change material. Renew. Sustain. Energy Rev..

[23] Fukui Y., Maruyama T., Iwamatsu Y., Fujii A., Tanaka T., Ohmukai Y., Matsuyama H. (2010). Preparation of monodispersed polyelectrolyte microcapsules with high encapsulation efficiency by an electrospray technique. Colloids Surf. Physicochem. Eng. Asp..

[24] Zhang L., Huang J., Si T., Xu R.X. (2012). Coaxial electrospray of microparticles and nanoparticles for biomedical applications. Expert Rev. Med. Devices.

[25] Bock N., Dargaville T.R., Woodruff M.A. (2012). Electrospraying of polymers with therapeutic molecules: State of the art. Prog. Polym. Sci..

[26] Enayati M., Chang M.-W., Bragman F., Edirisinghe M., Stride E. (2011). Electrohydrodynamic preparation of particles, capsules and bubbles for biomedical engineering applications. Colloids Surf. Physicochem. Eng. Asp..

[27] Zhang S., Campagne C., Salaün F. (2019). Influence of Solvent Selection in the Electrospraying Process of Polycaprolactone. Appl. Sci..

[28] Gómez-Mascaraque L.G., Tordera F., Fabra M.J., Martínez-Sanz M., Lopez-Rubio A. (2019). Coaxial electrospraying of biopolymers as a strategy to improve protection of bioactive food ingredients. Innov. Food Sci. Emerg. Technol..

[29] Ghayempour S., Mortazavi S.M. (2013). Fabrication of micro–nanocapsules by a new electrospraying method using coaxial jets and examination of effective parameters on their production. J. Electrost..

[30] Salaün F., Vroman I. (2008). Influence of core materials on thermal properties of melamine–formaldehyde microcapsules. Eur. Polym. J..

[31] Salaün F., Devaux E., Bourbigot S., Rumeau P. (2010). Influence of the solvent on the microencapsulation of an hydrated salt. Carbohydr. Polym..

[32] Fredi G., Dire S., Callone E., Ceccato R., Mondadori F., Pegoretti A. (2019). Docosane-Organosilica Microcapsules for Structural Composites with Thermal Energy Storage/Release Capability. Materials (Basel).

[33] Roy J.C., Ferri A., Giraud S., Jinping G., Salaun F. (2018). Chitosan(-)Carboxymethylcellulose-Based Polyelectrolyte Complexation and Microcapsule Shell Formulation. Int. J. Mol. Sci..

[34] Roy J.C., Giraud S., Ferri A., Mossotti R., Guan J., Salaun F. (2018). Influence of process parameters on microcapsule formation from chitosan-Type B gelatin complex coacervates. Carbohydr. Polym..

[35] Yang Z., Peng H., Wang W., Liu T. (2010). Crystallization behavior of poly(ε-caprolactone)/layered double hydroxide nanocomposites. J. Appl. Polym. Sci..

[36] Cho J.-S., Kwon A., Cho C.-G. (2014). Microencapsulation of octadecane as a phase-change material by interfacial polymerization in an emulsion system. Colloid. Polym. Sci..

[37] Su W., Darkwa J., Kokogiannakis G. (2015). Review of solid–liquid phase change materials and their encapsulation technologies. Renew. Sustain. Energy Rev..

[38] Zhang S., Campagne C., Salaün F. (2019). Preparation of Electrosprayed Poly(caprolactone) Microparticles Based on Green Solvents and Related Investigations on the Effects of Solution Properties as Well as Operating Parameters. Coatings.

[39] Chen H., Zhao Y., Song Y., Jiang L. (2008). One-step multicomponent encapsulation by compound-fluidic electrospray. J. Am. Chem. Soc..

[40] Labbaf S., Ghanbar H., Stride E., Edirisinghe M. (2014). Preparation of multilayered polymeric structures using a novel four-needle coaxial electrohydrodynamic device. Macromol. Rapid Commun..

[41] Lee Y.H., Mei F., Bai M.Y., Zhao S., Chen D.R. (2010). Release profile characteristics of biodegradable-polymer-coated drug particles fabricated by dual-capillary electrospray. J. Control. Release.

[42] Cui L., Liu Z.-P., Yu D.-G., Zhang S.-P., Bligh S.W.A., Zhao N. (2014). Electrosprayed core-shell nanoparticles of PVP and shellac for furnishing biphasic controlled release of ferulic acid. Colloid. Polym. Sci..

[43] Kim W., Kim S.S. (2010). Multishell encapsulation using a triple coaxial electrospray system. Anal. Chem..

[44] Zhang C., Yao Z.C., Ding Q., Choi J.J., Ahmad Z., Chang M.W., Li J.S. (2017). Tri-Needle Coaxial Electrospray Engineering of Magnetic Polymer Yolk-Shell Particles Possessing Dual-Imaging Modality, Multiagent Compartments, and Trigger Release Potential. ACS Appl. Mater Interfaces.

[45] Chen C., Zhao Y., Liu W. (2013). Electrospun polyethylene glycol/cellulose acetate phase change fibers with core–sheath structure for thermal energy storage. Renew. Energy.

[46] Hu W., Yu X. (2012). Encapsulation of bio-based PCM with coaxial electrospun ultrafine fibers. RSC Adv..

[47] Chen C., Wang L., Huang Y. (2008). Morphology and thermal properties of electrospun fatty acids/polyethylene terephthalate composite fibers as novel form-stable phase change materials. Sol. Energy Mater. Sol. Cells.

[48] Moghaddam M.K., Mortazavi S.M., Khayamian T. (2015). Preparation of calcium alginate microcapsules containing n-nonadecane by a melt coaxial electrospray method. J. Electrost..

[49] Moghaddam M.K., Mortazavi S.M., Khaymian T. (2015). Micro/nano-encapsulation of a phase change material by coaxial electrospray method. Iran. Polym. J..

[50] Kamali Moghaddam M., Mortazavi S.M. (2015). Preparation, characterisation and thermal properties of calcium alginate/n-nonadecane microcapsules fabricated by electro-coextrusion for thermo-regulating textiles. J. Microencapsul..

[51] Yuan W.-J., Wang Y.-P., Li W., Wang J.-P., Zhang X.-X., Zhang Y.-K. (2015). Microencapsulation and characterization of polyamic acid microcapsules containing n-octadecane via electrospraying method. Mater. Express.

[52] Zhang S., Campagne C., Salaün F. (2020). Preparation of n-Alkane/Polycaprolactone Phase-Change Microcapsules via Single Nozzle Electro-Spraying: Characterization on Their Formation, Structures and Properties. Appl. Sci..

[53] Hwang Y.K., Jeong U., Cho E.C. (2008). Production of uniform-sized polymer core-shell microcapsules by coaxial electrospraying. Langmuir.

[54] Nie H., Dong Z., Arifin D.Y., Hu Y., Wang C.H. (2010). Core/shell microspheres via coaxial electrohydrodynamic atomization for sequential and parallel release of drugs. J. Biomed. Mater. Res. A.

[55] Davoodi P., Feng F., Xu Q., Yan W.C., Tong Y.W., Srinivasan M.P., Sharma V.K., Wang C.H. (2015). Coaxial electrohydrodynamic atomization: Microparticles for drug delivery applications. J. Control. Release.

[56] Gañán-Calvo A.M., López-Herrera J.M., Herrada M.A., Ramos A., Montanero J.M. (2018). Review on the physics of electrospray: From electrokinetics to the operating conditions of single and coaxial Taylor cone-jets, and AC electrospray. J. Aerosol Sci..

[57] Fu D., Su Y., Xie B., Zhu H., Liu G., Wang D. (2011). Phase change materials of n-alkane-containing microcapsules: Observation of coexistence of ordered and rotator phases. Phys. Chem. Chem. Phys..

[58] Illeková E., Miklošovičová M., Šauša O., Berek D. (2011). Solidification and melting of cetane confined in the nanopores of silica gel. J. Therm. Anal. Calorim..

[59] Valo H., Peltonen L., Vehvilainen S., Karjalainen M., Kostiainen R., Laaksonen T., Hirvonen J. (2009). Electrospray encapsulation of hydrophilic and hydrophobic drugs in poly(L-lactic acid) nanoparticles. Small.

[60] Mochane M.J., Luyt A.S. (2012). Preparation and properties of polystyrene encapsulated paraffin wax as possible phase change material in a polypropylene matrix. Thermochim. Acta.

[61] Jenquin M.R., McGinity J.W. (1994). Characterization of acrylic resin matrix films and mechanisms of drug-polymer interactions. Int. J. Pharm..

[62] Dubernet C. (1995). Thermoanalysis of microspheres. Thermochim. Acta.

